# Neural responses associated with attentional engagement and disengagement from threat in high socially anxious children: Evidence from temporal-spatial PCA

**DOI:** 10.1371/journal.pone.0261172

**Published:** 2022-01-14

**Authors:** Erika Wauthia, Fabien D’Hondt, Wivine Blekic, Laurent Lefebvre, Laurence Ris, Mandy Rossignol

**Affiliations:** 1 Laboratory of Cognitive Psychology and Neuropsychology, Faculty of Psychology and Educational Sciences, University of Mons, Mons, Belgium; 2 Interdisciplinary Research Center in Psychophysiology and Cognitive Electrophysiology, Mons, Belgium; 3 National Fund for Human Science Research, National Fund for Scientific Research (FNRS), Brussels, Belgium; 4 Univ. Lille, INSERM U1172, CHU Lille, Centre Lille Neuroscience & Cognition, Lille, France; 5 CHU Lille, Clinique de Psychiatrie, Unité CURE, Lille, France; 6 Centre national de ressources et de résilience Lille-Paris (CN2R), Lille, France; 7 Neurosciences Laboratory, Faculty of Medicine, University of Mons, Mons, Belgium; National Institutes of Health, UNITED STATES

## Abstract

**Background:**

Cognitive models indicated that social anxiety disorder (SAD) would be caused and maintained by a biased attentional processing of threatening information. This study investigates whether socially anxious children may present impaired attentional engagement and disengagement from negative emotional faces, as well as their underlying event-related potential responses.

**Methods and findings:**

Fifteen children with high levels of social anxiety (HSA; 9 boys; mean age = 9.99y; SD = 1.14) and twenty low socially anxious children (LSA; 16 boys; mean age = 10.47y; SD = 1.17) participated in a spatial cueing task in which they had to detect targets following neutral/disgusted faces in a valid or invalid location. No group effect was reported on reaction times [p>.05]. However, electrophysiological data showed lower P3a amplitude in HSA children compared with the LSA group when processing facial stimuli. They also reported larger N2 amplitudes for valid-disgusted targets and a larger P3a amplitude for the invalid-disgusted ones.

**Conclusion:**

In terms of electrophysiological data, our results validated, the hypothesis of attentional disengagement difficulties in SAD children. We also confirm the idea that high levels of social anxiety are associated with cognitive control impairments and have a greater impact on the processing efficiency than on the performance effectiveness.

## 1. Introduction

Social anxiety disorder (SAD) refers to a condition in which individuals experience fear when they are performing a specific social task under the scrutiny of others [1]. SAD affects up to 10% of the pediatric population [2–5]. It can develop as early as 8 years old [6] and is associated with several negative consequences on social, academic and familial life, that can predict a more severe and persistent form of the disorder in adulthood [7, 8].

Recent research on the cognitive factors of SAD highlighted that the onset and maintenance of the disorder may arise from a biased attentional system in favor of threat-related stimuli such as human faces showing signs of disapproval or criticism [9–13]. Three mechanisms acting at different information processing stages are likely to contribute to this enhanced attentional processing, known as attentional bias (AB) for threat [14]: (i) a lower detection threshold for threatening stimuli resulting in an initial hypervigilance; (ii) a sustained attention to threatening cues leading to attentional disengagement impairments and to a stronger cognitive elaboration of the threat; (iii) a subsequent attentional avoidance when the threat is displayed for longer periods [15, 16]. The most widely used paradigm to assess AB is the visual dot-probe task [17] in which paired stimuli (e.g. a threat-neutral word pair or an angry-neutral face pair) are presented simultaneously on a computer screen. The stimuli then disappear and are followed by a visual target (e.g. a dot), presented in the spatial location previously occupied by the threatening (valid condition) or the neutral (invalid condition) stimulus. AB for threat classically infer from shorter reaction times (RTs) to detect targets in the valid condition and by longer RTs to detect targets in the invalid condition [18].

The attentional control theory [19, 20] postulates that attentional control plays a central role in the occurrence of AB for threat in anxiety disorders and has been supported by behavioral [21, 22] and neuroimaging results [23, 24]. Attentional control is known to be critical for inhibition and attentional shifting [19, 20, 25]. Inhibition enables the withstanding of interference from task-irrelevant stimuli or responses, while attentional shifting underlies attention flexibility abilities and the attentional focus on task-relevant stimuli of importance [20, 25]. Because of the inhibition and shifting deficits, anxious individuals would present a less efficient top-down attentional control, leading to an imbalance between the goal-oriented and the stimulus-driven attentional systems. Consequently, anxious individuals would display a hyperactive stimulus-driven system associated with the facilitated detection of threatening cues and attentional engagement towards them.

While AB for threat has been well-documented regarding anxious adults [9], there are inconsistent results among children. On the one hand, there is evidence for attentional hypervigilance for threat in children with various types of anxiety disorders (for a review, see [26]). In particular, Roy et al. (2008) [27] demonstrated a greater AB for angry faces in children aged between 7 and 18 years old and suffering from a generalized anxiety disorder, SAD and/or separation anxiety disorder, in comparison to their healthy peers. Results from the study conducted by Waters and colleagues (2011) [28] on socially anxious children aged between 5 and 12 years old revealed an AB for angry faces only in children with high symptom severity, while children with low symptom severity showed a bias away from the threat. On the other hand, several studies failed to show any AB for threat in children suffering from various types of anxiety disorders [9, 29]. The model proposed by Eysenck et al. (2007) also posits that group differences between anxious and non-anxious individuals for threat processing may not appear at behavioral level because anxiety acts more on processing efficiency (i.e. the amount of cognitive efforts needed) than on performance effectiveness (i.e. the performance quality). In other words, anxious individuals may recruit more neural resources during task performance to compensate for their reduced attentional control and their inhibition impairments, and finally behave as non-anxious individuals. Therefore, behavioral measures that focus on performance effectiveness may be not sensitive enough to assess efficiency deficit and detect AB. The application of this model to children suffering from social anxiety could therefore justify the inconsistencies observed in the literature.

The event-related potentials (ERP) technique seems particularly relevant to measure these two dimensions of efficiency and effectiveness. Indeed, because of its high temporal resolution, it allows to examine the time course of cerebral activations and to infer on cognitive processing steps leading to behavioral performance. Accordingly, ERPs may allow disentangling whether AB for threat is due to a faster engagement for threat and/or to a difficulty in disengaging from threat [30, 31]. They can also identify whether anxiety influences information processing and examine whether compensatory mechanisms appear, even in the absence of group differences between anxious and non-anxious individuals at a behavioral level. Finally, this noninvasive method is well-suited to experimentation with young children [32].

Only a few studies used ERPs to assess the cerebral correlates of threat processing in children with SAD [33] or at high risk for SAD [34, 35] and results showed increased amplitudes in early and late ERP components. Notably, these studies found an enhancement of the P1 amplitude in response to angry faces in these children. The P1 is a positive deflection that peaks approximately 90–110 ms after the occurrence of the stimulus [36, 37]. It is elicited from the extrastriate areas of the visual cortex when a stimulus is detected [38] and indexes early, pre-attentive processes [39]. Its amplitude increases for attended stimuli and can be modulated by top-down attention processes [40]. The results obtained by Bechor and colleagues (2019) [33] and by Pollak and Tolley-Schell (2003) [34] suggest that anxious children may devote more attentional resources associated with early orienting processes towards threatening facial stimuli. Bechor et al. (2019) [33] also found a larger N170 amplitude in anxious children by comparison to a control group, particularly when exposed to threatening cues. The N170 is a negative early component peaking around 140 and 180 ms after stimulus onset at occipito-parietal sites [41, 42] and is related to the processing of facial structures [41, 43]. The third interesting ERP component to investigate in anxious children would be the N2. The N2 is a fronto-central negativity generated by the anterior cingulate cortex (ACC) and the orbito-frontal cortex [44, 45] which occurs 200–300 ms post-stimulus and reflects cognitive control [46] and conflict monitoring [24, 47]. As for that component, only Thai, Taber-Thomas and Pérez-Edgar (2016) [35] reported that, during facial cues processing, increased amplitudes were associated with attentional bias as measured through RTs. Interestingly, these authors also found that only non-anxious children had higher P1 amplitudes for targets replacing angry faces, suggesting that non-anxious children may also be prone to show a hypervigilance towards threat-cued locations. Finally, ERP studies conducted in pediatric populations also investigated two higher-order cognitive ERP components, namely the P3a, which is associated with the detection and involuntary attentional orienting towards an incoming distractor stimuli [48–51] and the P3b, which is thought to reflect involuntary switching of attention towards distractors and their inhibition, according to the specific contents to the task at hand [50, 52]. Both components peak between 300 and 500 ms after stimulus onset [53] but while the P3a is thought to be maximal at fronto-central sites, the P3b is maximal at centro-parietal sites [49, 52, 54]. Bechor and colleagues (2019) [33] found reduced P3b in response to all facial stimuli in a group of anxious children aged between 8 and 12 by comparison to a control group. Authors have interpreted this result as indexing a poorer attentional control in pediatric anxiety. Furthermore, Pollak and Tolley-Schell [34] found, in children at higher risk of anxiety disorder, larger P3b amplitudes regarding the processing of targets following invalid angry faces comparing with invalid happy faces, suggesting an increased need for resources to disengage attention from a location previously occupied by a threat.

However, two major difficulties have been outlined in studies measuring ERP in pediatric populations. First, the child’s brain is still developing and components may present particularities in terms of topography or temporality, which complicates their identification and functional understanding [55–57]. Secondly, as outlined by Kujawa, Weinberg, Hajcak and Klein (2013) [58], EEG data in children are more likely to include noise and artifacts. These two considerations point to the need for a more refined analysis method to obtain more consistent ERP data [58]. In this perspective, temporal-spatial PCA have been proposed as a useful and more reliable measurement data for separating sources of variability in components and differentiating latent components from unsystematic sources of noise in adults [59, 60] and children [60, 61].

The aim of the current study was to assess the cognitive correlates of the processing of emotional stimuli in children with social anxiety and to provide support for the phenomenon of AB by recording evoked potentials and subjecting the data to a temporal-spatial PCA. To this end, we used an emotional spatial-cueing task, in which children had to detect a target preceded by a single cue presented in the left or the right hemifield. Cues may orient attention toward a valid location (when the target will appear on the same hemifield) or an invalid one (when targets appear on the opposite hemifield). Unlike the visual dot-probe task using a pair of cues, this paradigm allows to postulate that participants focus their attention on the single cue, and allows distinguishing between attentional engagement and disengagement processes [62]. Moreover, we chose to use faces expressing disgust instead of anger because disgust faces convey a negative evaluation of rejection and avoidance and are rated even more negatively than angry faces by anxious individuals [63–67].

We hypothesized that all children would display an attentional preference for disgusted faces by comparison to neutral faces because of the increased arousal of these kinds of stimuli. However, we also postulated that this effect would be more prominent in HSA children, since social anxiety increases attention towards social threat. Following the main assumptions of the attentional control theory, we postulated that more group differences were to be observed at the electrophysiological level than at the behavioral level because of the supposed greater impact of high levels of anxiety on processing efficiency than on performance effectiveness. In other words, we did not expect to find significant group differences on the RTs associated with the processing of targets, but we predicted to observe such differences on the ERP (e.g. increased amplitudes) during both the processing of threatening facial cues and targets following them.

## 2. Method

### 2.1 Participants

Fifteen children (6 girls) aged between 8 and 12 years old (*mean age* = 9.99; *SD* = 1.17) and presenting high levels of social anxiety were selected to participate in this study. We selected this age range because of evidence suggesting that more than 50% of socially anxious children develop their symptoms before 13 years old [68] and that symptoms can already be seen in children as young as 8 years old [6]. Children were included in the high social anxiety (HSA) group if they showed a score of 18 or higher at the Social Phobia and Anxiety Inventory for Children (SPAI-C; [69]). They were paired to twenty low socially anxious (LSA) children (4 girls; *mean age* = 10.47; *SD* = 1.14), reporting a score below 18 at the SPAI-C. All children were also assessed for state and trait anxiety using the State-Trait Anxiety Inventory for Children (STAI-C; [70]). The sample characteristics are presented in Table 1. All children were free from learning, neurologic or other psychiatric disorders as assessed with the Child Behavior Checklist [71, 72]. The ethical board of the Faculty of Psychology and Educational Sciences of the University of Mons approved this study. All participants and their legal guardian gave written informed consent. The work described has been carried out in accordance with the Code of Ethics of the Declaration of Helsinki. Children received a €20 gift-card for their participation.

**Table 1:** Demographic and psychopathological data for the LSA and HSA groups (** p-values < .01; * p-values < .05) (M = male; F = female).

	LSA		HSA		Group comparison
	M	SD	M	SD	p
Gender (M/F)	16/4		9/6		.201
Age (years)	10.47	1.14	9.99	1.17	.233**
State Anxiety	39.30	3.34	38.87	2.47	.676**
Trait Anxiety	34.31	7.87	39.33	5.96	.049**
Social Anxiety	*9.67	4.84	21.29	2.75	.001**

### 2.2 Task and materials

The emotional spatial-cueing task was adapted from the protocol used by Bar-Haim, Morag and Glickman (2011) [30]. The task was administered using E-Prime 2.0 software (Psychology Software Tools, Pittsburgh, PA) on an Asus X756U system (screen of 17 inches) in the Cognitive Psychology and Neuropsychology research laboratory of the University of Mons. Each trial began with a fixation cross appearing at the center of the screen for 500 milliseconds. Then, two rectangular frames (600 mm X 1000 mm) appeared on the right and the left side of the fixation cross for 500 milliseconds, and one of them showed a face depicting either a disgusted or a neutral expression. The pictures shown were faces of 10 different children (5 boys and 5 girls) from the Radboud Faces Database [73]. After the faces disappeared, a target (a star) (400 mm X 400 mm) appeared at the center of one of the rectangles. In 50% of the trials, targets appeared in the previous location of the face (valid trials) and in the remaining 50%, targets appeared on the opposite side of the screen (invalid trials). Participants were told to indicate as fast as possible the location of the target by pressing the corresponding button of the mouse. Targets remained on the screen until a response was made. A new trial began 500 milliseconds after the target offset. Before the test, participants received 16 randomly presented practice trials, 4 per condition (Valid/Invalid Neutral; Valid/Invalid Disgust). The entire task comprised 512 trials, presented in 8 blocks of 64 trials each. Within each block, 50% of the trials presented disgusted faces and 50% presented neutral faces. Trials were presented in a randomized order.

### 2.3 Behavioral data

RTs for each response corresponded to the time between the presentation of the target and the button press. Only RTs for correct responses were analyzed. Trials with RT longer than 2000 milliseconds and shorter than 150 milliseconds and then those with RT higher than 2.5 SDs above or lower than -2.5 SDs below the participants’ mean were discarded in order to reduce the influence of outliers. Individual mean RTs were computed for each experimental condition on the remaining trials (93.88% of the data).

### 2.4 EEG acquisition, preprocessing and analysis

EEG data were recorded at a sampling rate of 500 Hz (V-Amp, Brain Products, GmbH, Munich, Germany: 0–500 Hz bandwidth, 24-bit A/D conversion) from 64 electrodes sites (AF3, AF4, AF7, AF8, AFz, C1, C2, C3, C4, C5, C6, CP1, CP2, CP3, CP4, CP5, CP6, CPz, Cz, F1, F2, F3, F4, F5, F6, F7, F8, FC1, FC2, FC3, FC4, FC5, FC6, FCz, Fp1, Fp2, FT10, FT7, FT8, FT9, Fz, O1, O2, Oz, P1, P2, P3, P5, P6, P7, P8, PO3, PO4, PO7, PO8, POz, Pz, T7, T8, TP10, TP7, TP8, and TP9), with an actiCap acquisition system arranged in a standard 10–20 layout (Brain Products, GmbH, Munich, Germany). One electrode (P4) has been excluded from further analyzes due to poor functioning during data acquisition. The reference electrode was located at FCz throughout the recording. The ground electrode was placed on the forehead between Fp1 and Fp2. Electrode impedances were kept below 10 kΩ. Off-line analysis was performed using Brain Vision Analyzer 2 software (Brain Products, GmbH, Munich, Germany). We applied a band-pass IIR filter (0.1 to 30 Hz) and then, to correct eye blink artifacts, we used a semi-automatic ocular correction function implemented in the Analyzer 2.0 that used a simplified version of the independent component analysis (ICA) to correct ocular artifact specifically in the EEG signal [74]. This method has been proven to be useful to separate neural activity from muscle and blink artifacts in EEG data (e.g. [75]) and has been shown to present several advantages compared to other artifact removal methods (see [74]). All data were re-referenced to the average of all scalp electrodes. Finally, both for faces and targets processing, we created epochs by segmenting data from 200 milliseconds before and 500 milliseconds after stimulus onset. Epochs were baseline corrected using the mean voltage calculated from the 200 milliseconds preceding the event. Each epoch of 700 milliseconds with at least one channel showing a maximal amplitude difference of 200 μV was excluded from further analysis. Stimulus-locked ERPs were averaged separately for faces and targets.

ERP averages for each participant in each experimental conditions were submitted to temporal-spatial principal component analyses (PCA) using the ERP PCA Toolkit, version 2.7 [76] and respected the published guidelines for applying PCA to ERP datasets [77, 78]. PCA analyses are used to extracts linear combinations of data that distinguish patterns of electro-cortical activity across all-time points and recording sites [58]. Separated analyses were conducted for faces and targets processing. Firstly, a temporal PCA was performed to capture the variance across time points and to maximize the initial separation of ERP components [79]. All-time points were used as variables, and we considered all subjects, conditions and recording sites as observations. Simulation studies previously conducted suggested that Promax rotations are the most effective for temporal analyses since they do not force orthogonality amongst the components [76, 78]. Therefore, a Promax rotation was used, and following this first rotation, a parallel test [80] was conducted on the resulting Scree plot [81], in which the Scree of our dataset is compared to a Scree plot derived from a fully random dataset. The number of factors retained is thus based on the largest number of factors that account for a greater proportion of variance of the fully random data set (see [76] for more information). Based on this criterion and on the resulting Scree plots [81], seventeen temporal factors were extracted for faces processing (total variance explained = 97.3%), and eighteen temporal factors were extracted for targets processing (total variance explained = 91.5%). Each temporal factor may be considered to be a virtual epoch and can be described both by its factor loading (which describes the time course of that factor) and its factor scores (which give that factor’s value for each combination of subject, picture type, and recording site). Spatial information is preserved by temporal PCA; scalp topography can be reconstructed for any time point, subject, and condition by multiplying the corresponding electrode scores by the factor loading and standard deviation [82].

A spatial PCA was then performed on each of the temporal factors to synthesize the spatial dimensions of the dataset. Since simulation studies suggest that Infomax rotations are the most effective for spatial analyses (52, 54), an Infomax rotation was used for faces and targets processing separately. Recording sites were used as variables and all subjects, conditions and temporal scores were used as observations, thus a spatial PCA was conducted on each temporal factor retained in the previous step. Based on the average Scree plots resulting from the parallel test [80], six spatial factors were extracted for each of the 17 temporal factors identified for faces processing (total variance explained = 83.7%), resulting in 102 unique combinations of factors for faces processing. Following the same procedure for targets processing, six spatial factors were extracted for each of the 17 temporal factors identified (total variance explained = 84.3%), resulting in 108 combinations of factors. The covariance matrix and Kaiser normalization were used for each PCA [83]. To assess timing and spatial voltage distributions directly, we translated all factors back into voltages (μV) by multiplying factor scores by their corresponding loadings and standard deviations; in this way, both the time course and scalp topography of the electrocortical activity captured by that temporospatial factor combination can be directly assessed [59].

The temporal-spatial factors that accounted for at least 0.5% unique variance, i.e. 40 factors for faces processing and 39 factors for targets processing, were subjected to a robust ANOVA [84–86] to determine which components were significantly modulated by experimental conditions. As recommended by Dien and colleagues (2005), a visual inspection of the waveforms associated with each combination of factors was used to select those that corresponded the most to ERP components relevant to the paradigm used. Accordingly, and following the literature on the electrophysiological correlates of AB in anxious children [33–35], we considered as being representative for the P100, a maximal relative positivity recorded at occipital electrodes at ∼100 to 150 ms after stimulus onset. We will consider for the N170, a negative temporal deflection occurring and for the N200, a fronto-central negativity, peaking between 150 and 230 ms and 200 and 300 ms following stimulus onset, respectively. The P3a would be associated to a positivity located at fronto-central sites and peaking at ∼300 to 400 ms after stimulus onset; and for the P3b, a positivity located at parieto-occipital sites and peaking at ∼300 to 400 ms after stimulus onset.

### 2.5 Statistical analyses

Comparisons between groups were performed on anxiety (social, trait and state anxiety) characteristics using: (i) Wilcoxon rank-sum tests or independent t-tests for quantitative variables when the distributions of these variables were significantly different from normal or not, respectively; (ii) Pearson chi-square tests for qualitative variables. The normality of the distribution of quantitative parameters was assessed with Shapiro–Wilk tests. For RTs, General Linear Model analyses were conducted with 2-Emotion (Disgust and Neutral) X 2-Validity (Valid, Invalid) X 2-Visual Field (Left, Right) as within-subject factors and Groups (HSA, LSA) as the between-subjects factor. All the analyses have been performed using the software IBM SPSS Statistics for Windows (Version 21, Armonk, NY, IBM Corp.). The significance level was set at p < .05 (two-tailed) throughout the analyses.

Regarding ERP data, to avoid the biasing effects of non-normality, (co)variance heterogeneity between groups, and to reduce Type I errors [87], robust analyses of variance (ANOVA) were conducted on the selected temporal-spatial factors using the ERP PCA Toolkit [76, 85]. The seed for the number generation was set at 1000, and the number of iterations used for bootstrapping was 4,999 [77, 88]. Given potential variability in *p* values using this approach [84], simulations were run 11 times, with median *p* values reported, and only results in which the median *p* value plus 2 standard deviations remained below 0.05 were considered significant [84]. Robust ANOVA tests are indicated by “*TWJt/c*,” and the interpretation of this statistic and resulting p values are identical to a conventional ANOVA. Further information about robust ANOVA tests can be found at Dien (2010) [76].

A 2-Emotion (Disgust and Neutral) X 2-Visual Field (Left and Right) X 2-Group (HSA and LSA) analysis was conducted on temporal-spatial factors associated with faces processing. A 2-Emotion (Disgust and Neutral) X 2-Visual Field (Left and Right) X 2-Validity (Valid and Invalid) X 2-Groups (HSA and LSA) analysis was conducted on the temporal-spatial factors associated with targets processing. Only temporospatial factors for which statistically significant results were found are presented in the results section.

## 3. Results

### 3.1 Demographic and psychopathological data

Data are presented in Table 1. Groups did not significantly differ on sex ratio [*U* = 120; *p =* .201], age [*t*(33) = 1.22; *p =* .233] or state anxiety [*U* = 123.5; *p* = .370]. Social anxiety [*t*(33) = -8.32; *p* < .001] and trait anxiety [*t*(32) = -2.05; *p* = .049] was higher in the HSA group than in the LSA group.

### 3.2 Behavioral data

**Fig 1:**
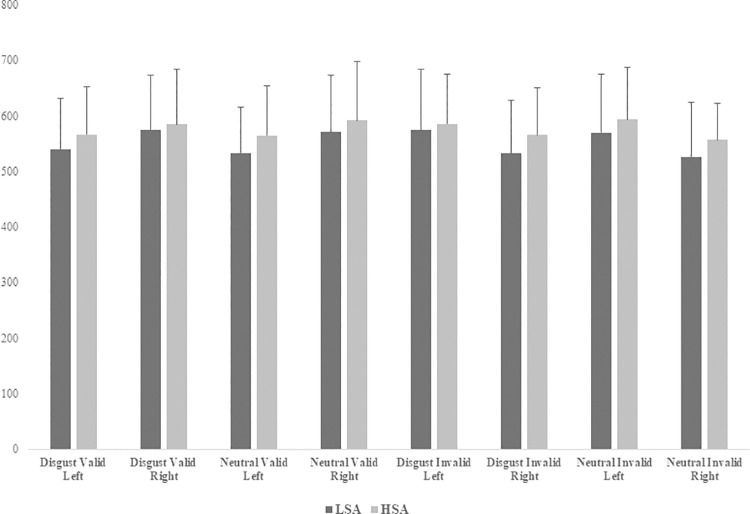
Mean reaction times (in milliseconds) obtained by each group of children in each experimental condition of the emotional spatial-cueing task.

Mean RTs obtained by each group in each experimental condition are presented in Fig 1. ANOVA conducted on RTs only revealed a significant interaction between Emotion and Validity [*F* (1,33) = 28.28; *p* < .001]. However, paired sample *T* tests conducted on this interaction failed to demonstrate significant differences between conditions [p-values > .05].

### 3.3 ERP data

#### 3.3.1 Faces processing

The original grand average waveforms for each face type and visual field are presented in Fig 1A. No significant effect was found on the factors corresponding to the N170 and the P3b components.

**Fig 2:**
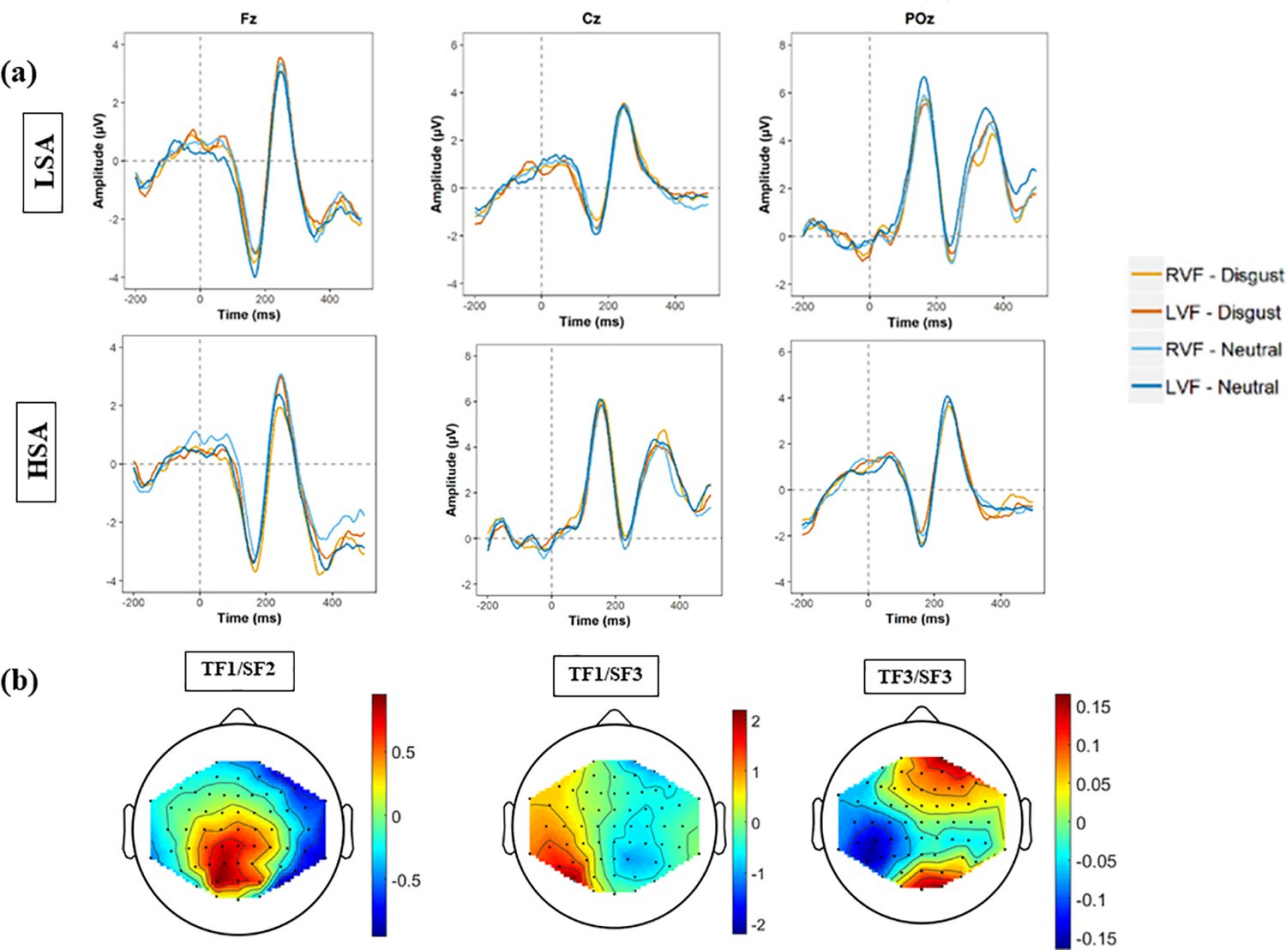
ERPs associated with faces processing. (a) Grand average ERPS prior to PCA analyses for the two types of faces (disgust and neutral) and the two visual fields (RVF = right visual field: LVF = left visual field) obtained at Fz, Cz, and POz; (b)Topographic maps for the temporal-spatial factors associated with faces processing.

*3*.*3*.*1*.*1 P1*. The two factors (TF1/SF2, TF1/SF3) representing the P1 component peaked at 154 milliseconds at parieto-occipital recording sites (Table 2 and Fig 2B). For the TF1/SF2 factor, the main effect of Emotion was significant [*T*_WJT/C_ (1.0, 30.0) = 5.41; *p* = .029], showing a higher amplitude for neutral faces [*M* = 1.93] than for disgusted faces [*M* = 0.91]. The main effect of EFE was also significant for the TF1/SF3 factor [*T*_WJT/C_ (1.0, 20.1) = 6.12; *p* = .025], again showing an increased amplitude for neutral [*M* = 2.64] compared to disgusted faces [*M* = 2.15].

**Table 2:** PCA factor combinations selected for further analysis on faces and targets processing.

Corresponding ERP component	Temporal-spatial factor combination	Variance explained	Temporal loading peak (ms)	Spatial loading peak	Polarity
Faces P100	TF1/SF2	31.9	154	PO8	Positive
	TF1/SF3	28.4	154	PO7	Positive
Faces P3a	TF3/SF3	2.5	382	FP2	Positive
Targets P100	TF2/SF1	48.7	154	PO8	Positive
	TF2/SF2	18.8	154	PO7	Positive
Targets N2	TF4/SF2	21.9	288	AF8	Negative
	TF4/SF4	6.1	288	PO7	Negative
Targets P3a	TF3/SF2	22.5	340	F7	Positive
	TF3/SF3	14.9	340	PO7	Negative

*3*.*3*.*1*.*2 P3a*. The TF3/SF3 factor was associated with the temporal window of the P3a, peaking at 382 milliseconds at frontal recording sites (Table 2 and Fig 2B). The main effect of Group was significant [*T*_WJT/C_ (1.0, 27.5) = 4.63; *p* = .002] with a greater amplitude in the LSA group [*M* = 0.75] than in the HSA group [*M* = -0.43].

#### 3.3.2 Targets processing

**Fig 3:**
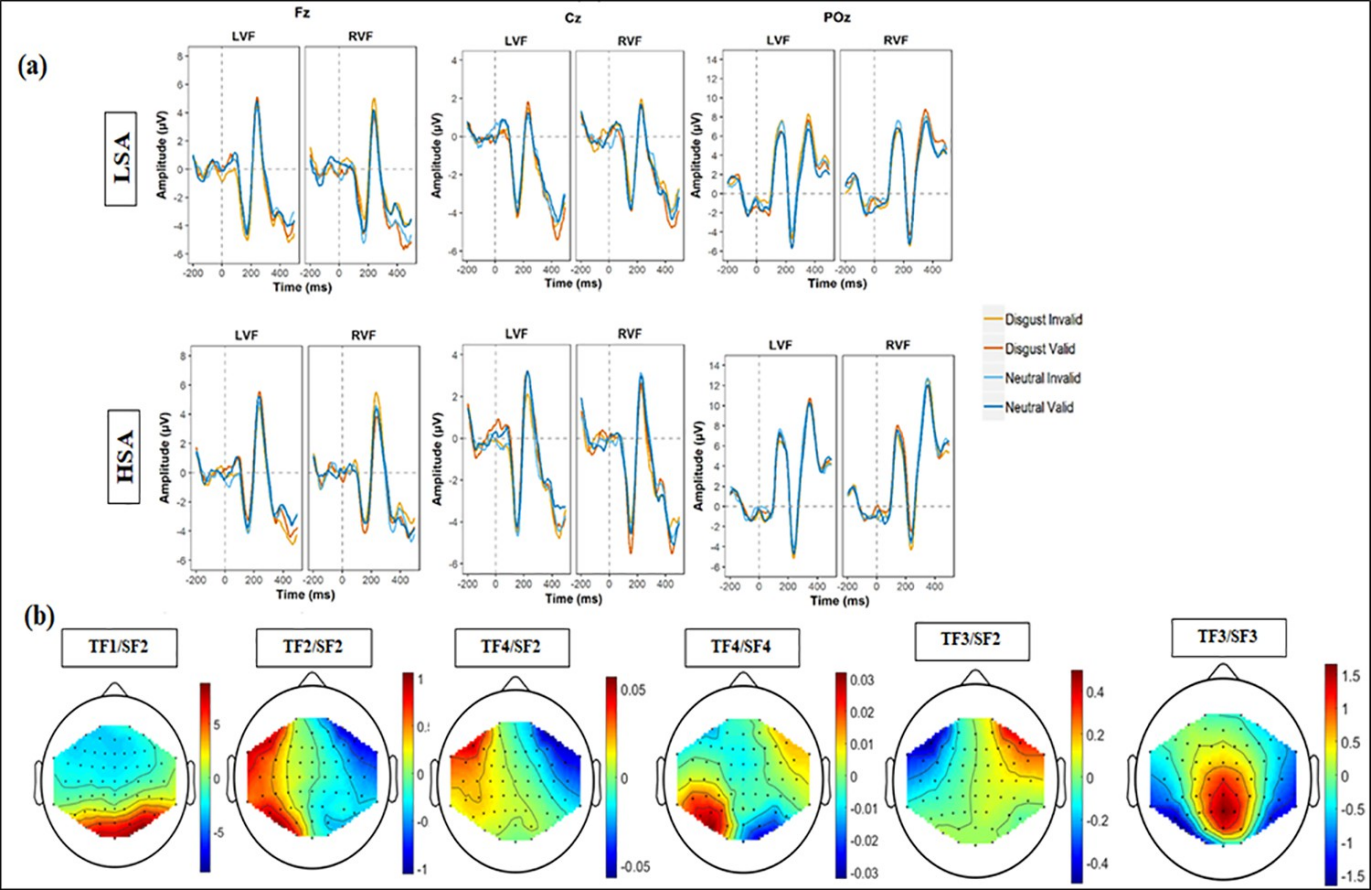
ERPs associated with targets processing. (a) Grand average ERPS prior to PCA analyses for the two types of faces (disgust and neutral) and the two validity conditions (valid and invalid), and the two visual fields (RVF = right visual field: LVF = left visual field) obtained at Fz, Cz, and POz; (b)Topographic maps for the temporal-spatial factors associated with targets processing.

The original grand average waveforms for each face type and visual field are presented in Fig 3A.

*3*.*3*.*2*.*1 P1*. The two factors associated with the P1 component (TF2/SF1, TF2/SF2) peaked at 154 milliseconds at parietal and parieto-occipital recording sites (Table 2 and Fig 3B). The interaction between Emotion and Validity was significant for the TF2/SF1 factor [*T*_WJT/C_ (1.0, 24.9) = 5.45; *p* = .030]. This interaction could be decomposed in a significant effect of Validity for targets following neutral faces only [*T*_WJT/C_ (1.0, 25.5) = 6.17; *p* = .023], showing an increased P1 amplitude for invalid-neutral [*M* = 9.54] compared to the valid-neutral targets [*M* = 8.91]. For the TF2/SF2 factor, there was a significant main effect of Emotion [*T*_WJT/C_ (1.0, 18.7) = 8.22; *p* = .010] with higher amplitudes for targets following neutral faces [*M* = 1.43] compared to disgusted faces [*M* = 1]. A main effect of Validity was also found on this factor [*T*_WJT/C_ (1.0, 29.8) = 29.11; *p* < .001], reflecting a greater P1 amplitude for targets in the valid [*M* = 1.51] than in the invalid condition [*M* = 0.93]. The interaction between Emotion and Validity was also significant for the TF2/SF2 factor [*T*_WJT/C_ (1.0, 24.9) = 15.66; *p* = .004]: amplitude was higher for targets in the disgust-valid [*M =* 1.05] condition compared to the neutral-valid condition [*M =* 0.90; *T*_WJT/C_ (1.0, 19.3] = 15.09; *p* = .004]. A significant interaction between Emotion and the Visual Field was found on the TF2/SF2 factor [*T*_WJT/C_ (1.0, 27.8) = 16.79; *p* < .001] highlighting that, when the target was presented on the left side of the screen, the P1 amplitude was increased for neutral [*M* = 0.12] compared to disgusted faces [*M* = -0.84; *T*_WJT/C_ (1.0, 21.7) = 18.61; *p* < .001].

*3*.*3*.*2*.*2 N2*. The two factors associated with the N2 were negative components peaking at 288 milliseconds at fronto-parietal (TF4/SF2) and parieto-occipital (TF4/SF4) recording sites (Table 2 and Fig 3B). For the TF4/SF2 factor, there was a significant main effect of Emotion [*T*_WJT/C_ (1.0, 26.9) = 10.98; *p* = .006] showing more negative amplitudes for targets following neutral faces [*M =* 0.53] than for targets following disgusted faces [*M =* -0.26]. The interaction between Emotion and Validity was significant for the TF4/SF2 factor [*T*_WJT/C_ (1.0, 29.5) = 39.99; *p* < .001], reflecting, in the valid condition, an increased amplitude for targets following disgusted faces [*M* = -0.35] in comparison to targets following neutral faces [*M* = 1.80; *T*_WJT/c_ (1.0, 24.9) = 8.91; *p* = .005].

For the TF4/SF4 factor, a significant interaction between Emotion and Validity was found [*T*_WJT/c_ (1.0, 28.7) = 10.65; *p* = .005] and indicated a wider N2 amplitude for disgusted faces [*M* = -0.12] than for neutral faces [*M* = 0.45] in the valid condition only [*T*_WJT/c_ (1.0, 24.9) = 8.91; *p* = .005]. The interaction between Emotion and Visual Field was significant [*T*_WJT/c_ (1.0, 27.4) = 4.60; *p* = .038] and revealed that, for targets presented on the left side of the screen, N2 amplitude was bigger for those following disgusted faces [*M* = -0.90] than for neutral faces [*M* = -0.42]. Finally, there was a significant interaction between Group, Emotion and Validity for the TF4/SF4 factor [*T*_WJT/c_ (1.0, 28.7) = 21.18; *p* < .001]. For the children with HSA, the interaction between Emotion and Validity [*T*_WJT/c_ (1.0, 13.0) = 28.68; *p* < .001] was significant, showing a more negative amplitude for targets in the disgust-valid [*M =* -0.54] compared to the disgust-invalid condition [*M =* 0.17; *T*_WJT/c_ (1.0, 13.0) = 13.96; *p* = .006]. The opposite pattern was found for targets preceded by neutral faces, with a more negative amplitude in the neutral-invalid [*M =* -0.55] in comparison to the neutral-valid condition [M = -0.30; T_WJT/c_ (1.0, 13.0) = 14.37; *p* = .019]. The interaction between Emotion and Validity was not significant for the LSA group [*T*_WJT/c_ (1.0, 17.0) = 0.97; *p* = .33].

*3*.*3*.*2*.*3 P3a*. The TF3/SF2 factor was associated with the P3a component and peaked at 340 milliseconds at frontal recording sites (Table 2 and Fig 3B). Moreover, the TF3/SF3 factor, a negative deflection that peaked at 340 milliseconds at parieto-occipital sites, could be topographically considered as a negative counterpart of the P3a component.

There was a significant main effect of Emotion for the TF3/SF2 [*T*_WJT/c_ (1.0, 26.8) = 31.5; *p* < .001] indicating wider amplitudes for targets appearing after disgusted faces [*M* = 0.02] than for targets appearing after neutral faces [*M =* -0.82; T_WJT/c_ (1.0, 26.8) = 31.50; p < .001]. The main effect of Validity was also significant, with an enhanced amplitude for targets appearing in the invalid condition [*M =* 0.24] compared to the valid condition [*M =* -1.03; T_WJT/c_ (1.0, 23.2) = 46.68; *p* < .001]. There was a significant interaction between Emotion and Validity [*T*_WJT/c_ (1.0, 28.5) = 41.53; *p* < .001] that showed larger amplitudes for targets in the neutral-valid [*M =* -2.02] than in the disgust-valid condition [*M =* -0.04; *T*_WJT/c_ (1.0, 28.0) = 61.41; p < .001]. No such effect was observed for targets in the invalid condition [*T*_WJT/c_ (1.0, 26.6) = 1.91; p = .017].

The interaction between Emotion and Visual Field was significant [*T*_WJT/c_ (1.0, 30.0) = 39.08; *p* < .001], indicating that, for targets presented on the left side of the screen, P3a amplitude was larger for targets following disgusted faces [*M* = 4.27] than neutral faces [*M* = 2.37; *T*_WJT/c_ (1.0, 25.8) = 51.03; *p* < .001]. Finally, the interaction between Emotion, Visual Field and Validity was also significant [*T*_WJT/c_ (1.0, 29.1) = 73.59; *p* < .001]. This interaction could be decomposed in a significant interaction between Emotion and Validity when targets are presented on the left side of the screen [*T*_WJT/c_ (1.0, 29.8) = 88.68; p < .001] which indicates larger P3a amplitude for targets in the disgust-valid [*M* = 3.70] than in the neutral-valid condition [*M* = -0.58; *T*_WJT/c_ (1.0, 27.4) = 93.84; *p* < .001]. No such effect was observed for targets in the invalid condition [*T*_WJT/c_ (1.0, 29.7) = 3.18; *p* = .084].

Analyses on the TF3/SF3 factor showed a significant interaction between Group, Emotion and Validity [*T*_WJT/c_ (1.0, 27.7) = 4.89; *p* = .036]. For children with HSA, there was a significant interaction between Emotion and Validity [*T*_WJT/c_ (1.0, 13.0) = 7.17; *p* = .021] showing lower amplitudes for targets in the disgust-valid [*M =* -2.53] than in the disgust-invalid condition [*M =* -2.07; *T*_WJT/c_ (1.0, 13.0) = 6.43, *p* = .021]. We found no significant effect of Validity for neutral faces [*p* = .07]. No significant effect was observed for disgusted and for neutral faces for LSA [*p*-values > .05].

## 4. Discussion

This study aimed to investigate the mechanisms underlying the attentional bias towards disgusted faces in socially anxious children. To this end, 15 children with HSA and 20 children with LSA, all aged between 8 and 12, performed an emotional spatial-cueing task requiring them to detect a target following either neutral or disgusted faces while their electroencephalograms were recorded.

In line with our expectations, no group differences were found on RTs which confirmed some studies previously conducted in children with various anxiety disorders [89–91]. However, some significant differences appeared between experimental groups on various ERP components. We found increased N2 amplitudes for targets following valid disgusted faces and increased P3a amplitudes for targets following invalid disgusted faces in HSA children and in comparison to neutral faces. Taken together, these results support the idea of preferential neural processing of threatening stimuli in children with HSA. More precisely, they suggest that the attention of socially anxious children would have been captured by the presentation of the disgusted faces. Therefore, when asked to process a following target, HSA children would have experienced difficulties and needed more effort to disengage their attention from the faces to detect and evaluate a target appearing on the opposite side of the screen correctly [9, 92]. We also found lower frontal P3a amplitudes in HSA children in comparison to LSA children that can be related to the less effective attentional control frequently reported in anxious children [19, 93, 94] that would lead them to devote less neural resources to the detection and evaluation of faces which are task-irrelevant [33].

Several other significant effects appeared on ERP components, and these were conflicting with our initial hypotheses. Indeed, we documented in all children increased P1 amplitudes for the processing of neutral faces, higher P1 amplitudes for targets following neutral faces in the valid conditions, and enhanced N2/P3a amplitudes for targets following neutral faces in the invalid conditions and in comparison with disgusted faces. The results of our study tend to show that 8 to 12-year-old children have an enhanced and sustained attentional engagement towards neutral faces and that the processing of neutral faces would have required an increased cognitive control [46] and conflict monitoring abilities [24, 47]. Specifically, here, the conflict arose from the fact that participants had to inhibit the cognitive process of the cue to detect the target appearing on the opposite side of the screen correctly. Consequently, results suggest that children’s attentional focus would have been maintained on the spot previously occupied by the neutral faces and that they experienced subsequent attentional disengagement difficulties from these. The observation of increased ERP amplitudes in response to neutral faces was somewhat contradictory to our hypotheses and quite surprising given the documented AB towards negative expressions in pediatric populations (e.g. [95]). However, an increased processing of neutral faces has already been demonstrated in both socially anxious adults [96–100] and children [101]. We may interpret these effects within the framework of the attentional control theory [19], which initially states that anxious individuals would display *global* attentional control impairments that would be inflated during the confrontation to negative facial expressions. As explained by the theory, these impairments would lead to an imbalance between a hyperactive bottom-up attentional system and a hypoactive top-down system. Therefore, we hypothesize that the hyperactivation of the amygdala would have led children to interpret neutral faces as conveying a threatening load [100, 102]. Furthermore, and in line with the works by Kagan et al. [103, 104], one may hypothesize that the arousal level conveyed by the ambiguity of neutral faces may be considered as threatening by all children because of their uncertainty. If neutral faces can be interpreted as threatening, our observations question the relevance of opposing neutral and negative faces to assess attentional biases towards threat.

The observation of such an attentional bias towards neutral in both LSA and HAS group can be understood from a developmental point of view. Indeed, attentional control has been shown to rely on the dorsolateral prefrontal cortex [23, 105], which undergoes one of the longest periods of development of any brain region [21, 106] and developmental variabilities have been evidenced in studies involving attentional control functions until the age of 12 [107, 108]. We may posit that the function would still be immature in our selected age range (8–12 years old). Therefore, all the children of our study would have presented immature attentional control abilities, resulting in hypervigilance and facilitated detection of neutrality and in disengagement difficulties from what they may have considered as threatening. However, unlike socially anxious children, with age, we postulate that LSA children will be progressively more able to inhibit and control the automatic processing of threat [109, 110], which stresses the need for longitudinal studies on these issues.

Our findings should be considered cautiously regarding a number of limitations. The first limitation concerns the relatively small sample size and the use of robust ANOVAs that may limit the statistical power of analyses, but which outlined the need for future studies on a larger population. The second concerns the age of the children investigated. Children from our sample were aged between 8 and 12 years old, which prevented us from investigating the modulation of effects with age and with the maturation of attentional control. Studies found developmental variabilities in tasks involving attentional control until the age of 12 [107, 108]. It would thus be interesting to replicate this study with older children. Thirdly, we investigated a sub-clinical sample and not children with clinical levels of SAD. Therefore, we could assume that more pronounced or additional effects could be revealed in clinical samples. Furthermore, the effects–or the lack of effect–on the early visual component such as the P1 may arise from differences in the low-level properties between stimuli. Therefore, it could be useful, in future research, to assess these low-level properties or to control them beforehand. Finally, the presentation duration of our stimuli (500 milliseconds) prevented us from evaluating the components associated with the later-stage of attentional processing such as the late positive potential (LPP); for which interesting results for anxious children have already been demonstrated (for a review, see [39]).

## 5. Conclusion

This study is the first to investigate the neural responses associated with threat processing in children with high social anxiety levels. Results showed that high levels of social anxiety in children was not associated with behavioral manifestation of AB which confirms one of the main assumptions of the attentional control theory [19, 20] stating that the effects of anxiety are higher on processing efficiency than on performance effectiveness. However, ERP data showed that high socially anxious children experience difficulties to disengage their attention from disgusted faces. Social anxiety appears to act as a filter on the later stages of the (still early) attentional processing of threatening social stimuli. Our data confirmed the presence of attentional control deficits in HSA children, as suggested by the lower P3a amplitudes in response to all emotional faces in this group. They also demonstrated that HSA children appears to commit more neural resources to obtain behavioral performances similar to the ones of LSA children, as suggested by the increased N2 and P3a amplitudes for targets associated with disgusted faces. This suggests the applicability of the distinction between the notions of performance effectiveness and processing efficiency made by the attentional control theory [19, 20]. Our research also allows to assume that the N2 and the P3a components should be considered in further studies as biomarkers of high levels of social anxiety, notably when investigating the efficacy of attentional training sessions designed to reduced anxiety levels. Finally, we hypothesized that the ambiguity conveyed by neutral faces leads children to devote more neural resources to perceive them and perhaps interpret them as threatening probably due to the immaturity of the cerebral regions implicated in attentional control abilities in this age range. Further studies should investigate AB towards neutral social stimuli.
